# Chordoma of the posterior mediastinum accompanied by synchronous lesion

**DOI:** 10.1590/0100-3984.2016.0059

**Published:** 2017

**Authors:** Bruno Niemeyer de Freitas Ribeiro, Edson Marchiori

**Affiliations:** 1 Instituto Estadual do Cérebro Paulo Niemeyer, Rio de Janeiro RJ, Brazil.; 2 Universidade Federal do Rio de Janeiro (UFRJ), Rio de Janeiro, RJ, Brazil.

**Figure 1:**
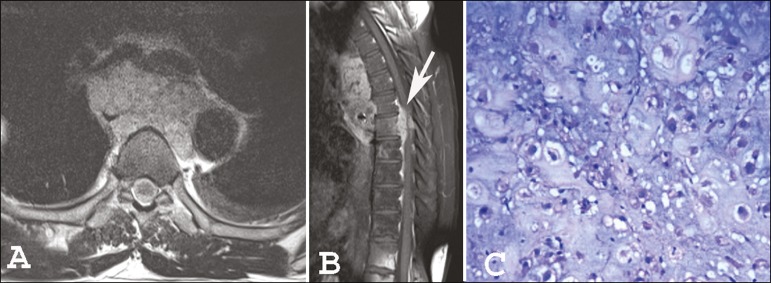
Magnetic resonance imaging scans: axial T2-weighted image (A) and contrast-enhanced sagittal T1-weighted image (B), showing a lesion affecting the posterior mediastinum and invading the vertebral canal (arrow in B). Histopathology (C) revealing physaliferous cells.

Dear Editor,

A 53-year-old male patient with a 3-month history of back pain presented with progressive paraparesis, although without loss of sphincter control. Magnetic resonance imaging (MRI) of the dorsal spine (Figures 1A and 1B) showed an expansile lesion with lobulated contours, involving the posterior mediastinum and extending to the vertebral canal, thus reducing the amplitude of the vertebral canal and compressing the medulla. A synchronic lesion of similar appearance, affecting the 12th dorsal vertebra, was observed. The histopathological study revealed large cells with vacuolated cytoplasm and partially vesicular nuclei (some demonstrating prominent nucleoli), with the appearance of physaliferous cells (from the Greek *physallis*, or bubble), consistent with a diagnosis of chordoma (Figure 1C).

Recent studies in the radiology literature of Brazil have highlighted the importance of imaging methods in improving the diagnosis of intrathoracic alterations^(1-5)^. Chordomas are slow-growing malignancies derived from primitive remnants of the notochord. They typically occur in the fifth and sixth decades of life^(6,7)^, with a slight predilection for males and preferential involvement of the sacrococcygeal region (50%), followed by the spheno-occipital region (35%), cervical spine, and lumbar spine, occurring only rarely in the dorsal spine and posterior mediastinum^(6-8)^. Symptoms often appear only after the lesion has reached large proportions, with local invasion affecting neurovascular structures. Local recurrence is common when complete resection was not possible.

The differential diagnoses of chordoma include metastases, chondrosarcoma, multiple myeloma, neurogenic tumors, among others. Although imaging methods help delineate the lesion, the diagnosis is made on the basis of the histopathological analysis^(7)^.

On MRI, most chordomas show isointense or hypointense signals in T1-weighted sequences, whereas they show hyperintense signals in T2-weighted and short-tau inversion-recovery sequences, reflecting their high water content, some lesions containing fibrous septa and therefore showing low signal intensity in T2-weighted sequences^(6-8)^. Gadolinium contrast enhancement tends to be moderate and heterogeneous^(6,8)^. Lesions are often accompanied by bone erosion, which was not observed in the case reported here. Recent studies have highlighted the use of diffusion-weighted imaging in the differentiation between chordomas and chondrosarcomas, reporting that the latter show higher apparent diffusion coefficients^(9,10)^.

In addition to an unusual site of involvement, our patient presented the peculiarity of a synchronous lesion. Although some authors have reported similar cases^(7,8,11,12)^, there is no specific criterion for differentiating between a multicentric chordoma and metastatic dissemination. We believe that our case could represent dissemination to the cerebrospinal fluid, because there was involvement of the vertebral canal.

The treatment of choice for chordoma is surgical resection with adjuvant radiotherapy, resulting in a disease-free period approximately 2.5 years longer than that achieved after surgical treatment alone^(7)^. Because chordoma is resistant to conventional radiotherapy, other modalities, such as stereotactic radiosurgery, are used. Chordoma does not respond well to chemotherapy, antitumor activity having been observed, in small studies, only with the use of imatinib mesylate^(13)^.

Albeit rare, a diagnosis of chordoma should be considered in patients with lesions affecting the posterior mediastinum. In addition, the possibility of synchronous lesions should be investigated in such patients.
